# PML Body Component Sp100A Is a Cytosolic Responder to IFN and Activator of Antiviral ISGs

**DOI:** 10.1128/mbio.02044-22

**Published:** 2022-11-16

**Authors:** Hongchang Dong, Wencheng Wu, Jingjing Li, Yilei Ma, Xiaomei Deng, Deyin Guo, Pei Xu

**Affiliations:** a The Centre for Infection and Immunity Studies, School of Medicine, Sun Yat-sen University, Shenzhen, People’s Republic of China; b Key Laboratory of Tropical Disease Control, Ministry of Education, Sun Yat-sen University, Guangzhou, People’s Republic of China; Griffith University

**Keywords:** PML, Sp100A, ISGs, antiviral responses, IFN

## Abstract

Promyelocytic leukemia protein (PML) bodies are implicated in one of the key pathways in the establishment of antiviral status in response to interferon (IFN), yet the molecular mechanisms bridging the cross talk remain elusive. Herein, we report that a major constitutive component of the PML body, Sp100A, is ubiquitously located in the cytosol of various cell types and is an immediate responder to multiple extracellular stimuli, including virus infection, IFN, epidermal growth factor (EGF), glial cell-derived nerve factor (GDNF), etc., signaling through the phosphatidylinositol 3-kinase (PI3K) pathway. IFN-β induces phosphorylation of Sp100A on Ser^188^, which fortifies the binding of Sp100A to pyruvate kinase 2 (PKM2) and facilitates its nuclear importation through the extracellular signal-regulated kinase 1/2 (ERK1/2)-PKM2-PIN1-importin axes. Blocking PI3K pathway signaling or interference with the ERK1/2-PKM2-PIN1-importin axes independently hampers nuclear translocation of Sp100A in response to IFN, reflecting a dual-regulation mechanism governing this event. In the nucleus, Sp100A is enriched in the promoter regions of essential antiviral interferon-stimulated genes (ISGs), such as those coding for IFI16, OAS2, and RIG-I, and activates their transcription. Importantly, nuclear importation of Sp100A, but not accumulation of a mutant Sp100A that failed to respond to IFN, during infection potently enhanced transcription of these antiviral ISGs and restricted virus propagation. These findings depict a novel IFN response mechanism by PML bodies in the cytosol and shed light on the complex sensing-regulatory network of PML bodies.

## INTRODUCTION

Promyelocytic leukemia protein (PML) nuclear bodies (NBs) (also known as ND10) are small (0.1 to 1 μm) membrane-less organelles that recruit an astonishing variety of permanently and transiently associated proteins (1–3). These intranuclear macromolecular multiprotein complexes are deeply involved in biological processes such as apoptosis, cell senescence, angiogenesis, oxidative stress response, and DNA damage response, etc., and are believed to play key roles in both receiving and processing regulatory signals and instructing corresponding cellular responses (4–9). An essential role of PML bodies in the establishment of an antiviral state in response to interferon (IFN) is evidenced by studies from at least three aspects. (i) Permanent components of PML bodies, including PML, Sp100 (i.e., speckled protein 100 kDa), and death domain-associated protein (Daxx), are IFN-stimulated proteins, and the intranuclear size and number of PML bodies are significantly induced upon IFN treatment (1, 10–12). Furthermore, in *PML* knockout mouse embryonic fibroblasts (MEFs), IFN treatment failed to mount an antiviral state comparable to that of wild-type MEFs (13–15). (ii) Various viruses have developed strategies to directly target PML body components and modify, dismiss, or disrupt PML body structures during infection (16–24). (iii) Accumulating evidence shows that components of PML bodies, including PML, hDaxx, Sp100, and ATRX, act as cellular restrictive factors of pathogens, including herpesviruses, HIV, vesicular stomatitis virus (VSV), severe acute respiratory syndrome coronavirus 2 (SARS-CoV-2), etc. (12, 14, 15, 25–33). However, the molecular networks mediating the cross talk between type I interferon (IFN) signaling and PML NBs and the position of PML bodies in the signaling hierarchy of type I IFN remain enigmatic.

Sp100 is a major constituent component of PML NBs. The *Sp100* gene encodes four isoforms, Sp100A, Sp100B, Sp100HMG, and Sp100C, through RNA splicing of a single primary transcript (2). Sp100 isoforms share an N terminus of 477 amino acids (aa) and differ in the length and sequence of their C termini (34–36). The link of Sp100 to the host antiviral response first emerged as two IFN responding elements in the promoter region of the *Sp100* gene (ISRE [ACTTTCACTTCTCT] and GAS [TTCCAGGAA] domains) were identified (1, 10) and is strengthened by the accruing evidence that various viruses employ diverse strategies to target or disperse Sp100 from PML NBs and that depletion of Sp100 protein by different strategies significantly facilitated viral progression (37–41). The latest research by our group showed that herpes simplex virus 1 (HSV-1)-infected HEp-2 cells increasingly secreted Sp100 into extracellular vesicles, which significantly inhibited virus infection in neighboring recipient cells, implying a critical role of cytosolic Sp100 in mediating antiviral responses (42).

In this investigation, we report that the PML NB constitutive component Sp100A is ubiquitously distributed in the cytosolic compartment of cells of various origins and is an immediate responder to IFN-β signaling in the cytoplasm. Cytosolic Sp100A was increasingly phosphorylated at Ser^188^ upon IFN treatment, which simultaneously fortified its binding affinity to PKM2 and promoted its nuclear translocation. This process relied on the activation of both the extracellular signal-regulated kinase 1/2 (ERK1/2)-PKM2-PIN1-importin axes and the phosphatidylinositol 3-kinase (PI3K) pathway, reflecting a dual-regulatory mechanism coaching this event. Furthermore, the whole-genome association pattern of Sp100A revealed that IFN-β caused enrichment of Sp100A in the promoter regions of several essential antiviral genes, such as those coding for RIG-I, IFI16, and OAS2, which positively correlated with their transcriptional activation. Importantly, the expression of Sp100A during VSV infection potently enhanced antiviral responses and significantly inhibited viral growth but not the expression of green fluorescent protein (GFP) or a mutant Sp100A that failed to translocate into the nucleus in response to IFN.

These findings not only have explained, for the first time, the detailed molecular mechanisms mediating the cross talk between IFN signaling and PML bodies but also serve as a delicate example illustrating how PML bodies receive variegated intra- and extracellular signals and translate them into epigenetic regulation of the cellular transcriptome.

## RESULTS

### Cytosolic Sp100A immediately translocated into the nucleus in response to virus infection and IFN-β.

**FIG 1:**
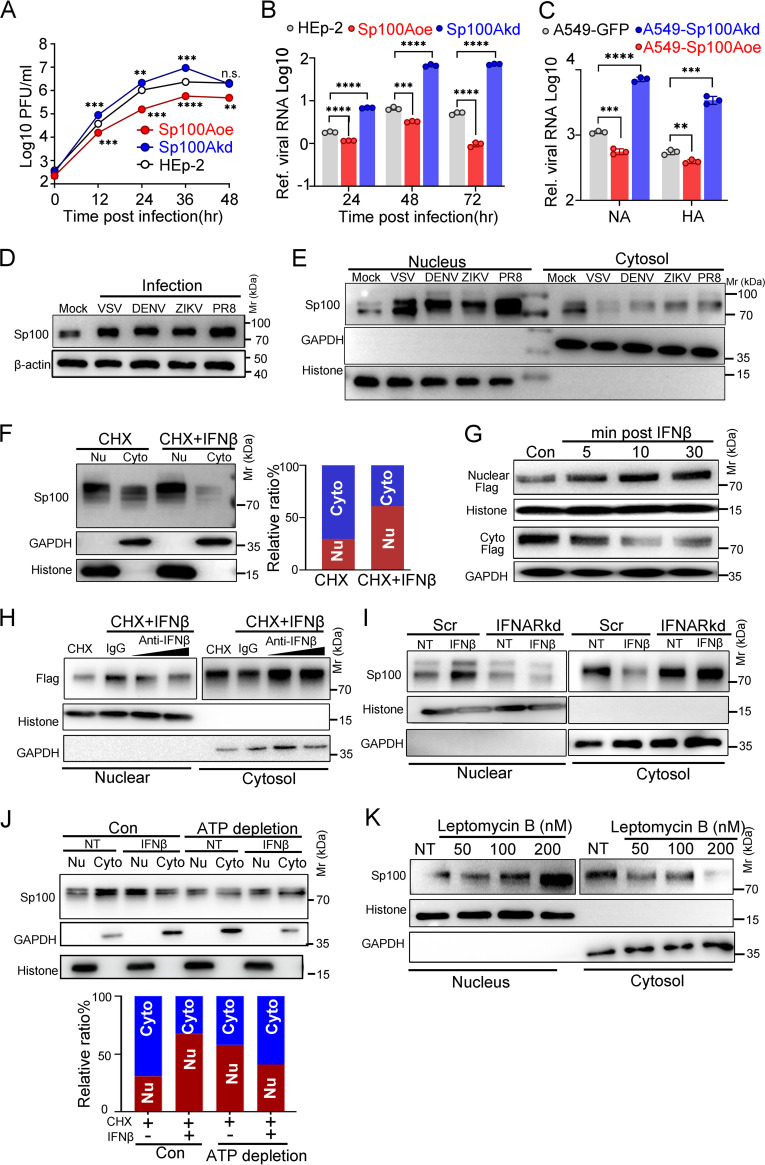
RNA virus infection induced nuclear recruitment of SP100A via IFN signaling. (A) Growth kinetics of VSV in Sp100Aoe, Sp100Akd, and HEp-2 cells at an MOI of 0.1. Statistical differences in virus growth in Sp100Aoe and Sp100Akd cells compared to that in HEp-2 cells are shown. (B) Viral RNA in the culture medium of DENV-2-infected HEp-2, Sp100Aoe, and Sp100Akd cells at 24, 48, and 72 hpi (MOI of 0.1) was quantified by qRT-PCR. (C) Viral RNA in influenza virus (PR8)-infected A549-GFP, A549-Sp100Akd, and A549-Sp100Aoe cells at 24 hpi (MOI of 0.1) was quantified by qRT-PCR using primers targeting the NA and HA fragments. (D and E) HFL1 cells were mock infected or infected with VSV-GFP, PR8, DENV-2, and ZIKV at an MOI of 5. At 6 hpi, whole-cell lysates were immunoblotted with antibodies reactive to Sp100 or β-actin (D), or cytosolic and nuclear lysates were loaded and immunoblotted with antibodies reactive to Sp100, GAPDH (glyceraldehyde-3-phosphate dehydrogenase), or histone (E). (F) HEp-2 cells were treated with CHX only or CHX plus IFN-β for 6 h. In the left panel, 40 μL out of 600 μL cytosolic lysate and 40 μL out of 200 μL nuclear lysate were loaded and immunoblotted with polyclonal anti-Sp100 antibody. Band density was quantified by ImageJ, and the nuclear/cytosolic Sp100 protein ratio was calculated and plotted as shown in the right panel. (G) Subcellular distribution of transfected NLS mutants in HEp-2 cells in response to IFN-β (1,000 U/mL) at the indicated time points was examined by immunoblotting (Sp100 band exposure times of 3 s for the nuclear portion and 3 s for the cytosolic portion). (H) HEp-2 cells were pretreated with CHX for 2 h and exposed to IFN-β (1,000 U/mL) mixed with IgG or anti-IFN-β antibody at 10 or 20 μg/mL for 2 h. The subcellular localization of endogenous Sp100 was determined by immunoblotting. (I) Cells stably expressing scramble shRNA (Scr) or shRNA targeting IFNAR (IFNARkd) were treated with CHX only (NT) or IFN-β plus CHX (IFNβ) for 2 h. The subcellular localization of endogenous Sp100 in response to IFN was measured (Sp100 band exposure times of 3 s for the nuclear portion and 10 s for the cytosolic portion in panels H and I). (J) Untreated or ATP-depleted HEp-2 cells were treated with CHX (NT) or CHX plus IFNβ (IFNβ) for 2 h. Forty microliters out of 600 μL cytosolic lysate and 20 μL out of 200 μL nuclear lysate were loaded. Band density was quantified and plotted. (K) HEp-2 cells were untreated or treated with increasing concentrations of leptomycin B. Subcellular fractions were immunoblotted with anti-Sp100 antibody (Sp100 band exposure times of 10 s for the nuclear portion and 30 s for the cytosolic portion). Two-tailed unpaired Student's *t* test was used to calculate *P* values in panels A, B, and C.

Prior studies have shown that Sp100A is a host restrictive factor of HSV-1 and conveys antiviral responses between infected and uninfected cells through extracellular vesicles (42). In this study, the molecular mechanism by which Sp100A restricts viral infection was investigated. Sp100A knockdown (Sp100Akd) and Sp100A-overexpressing (Sp100Aoe) cell lines were established based on HEp-2 cells or A549 cells (see Fig. S1A, B, and C in the supplemental material), and examination of the growth of VSV, dengue virus subtype (DENV-2), and influenza virus in these cells indicated that Sp100A had a general inhibitory effect on RNA viruses (Fig. 1A, B, and C). Further investigation showed that RNA virus infection not only induced an overall increase in endogenous Sp100 protein levels (Fig. 1 D and Fig. S1D) but also led to a significant increase in nuclear Sp100 accompanied by a decrease in cytosolic Sp100 in multiple cell lines examined (Fig. 1E and Fig. S1E and F). Transient transfection of individual Sp100 isoforms suggested that only Sp100A localized in both the nucleus and cytosol (Fig. S1G), and scrutiny of various human normal and cancer cell lines indicated a ubiquitous presence of cytosolic Sp100, matching the electrophoretic mobility of Sp100A in denatured polyacrylamide gels (Fig. S1H). As VSV induced a type I IFN response early in infection (Fig. S1I) and the Sp100 gene is a known interferon-stimulated gene (ISG) (1), whether type I IFN signals the nuclear translocation of Sp100A was investigated. As shown in Fig. 1 F, treatment with cycloheximide (CHX [protein synthesis blocker]) and IFN-β led to evident relocation of endogenous Sp100 from the cytoplasm to the nucleus. Importantly, nuclear translocation of ectopically expressed Sp100A was initiated as soon as 5 min postexposure to IFN-β and saturated at ~10 to 30 min posttreatment (Fig. 1G and Fig. S1J), implying that this molecular event was a primary response to IFN. Blockage of IFN signaling by an IFN neutralizing antibody or knockdown of the IFN-α/β receptor (IFNAR) abolished the nuclear importation of Sp100 (Fig. 1H and I). The detection of nuclear relocation of Sp100A in response to IFN in several other human cell lines expanded the generality of this phenomenon and shed light on its biological importance (Fig. S1K). It should be noted that although cytosolic Sp100 constitutes a previously neglected portion of the whole-cell level in HEp-2 cells (42), the nuclear translocation of cytosolic Sp100 (Cyto-Sp100) during virus infection was clear and prominent (Fig. S1L). As indicated in the figure legends, Cyto-Sp100 was loaded in extra amounts or exposed for a longer time in some figures as indicated.

10.1128/mbio.02044-22.2 FIG S1 Construction of Sp100A overexpression and knockdown cell lines and examination of nuclear translocation of Sp100A. (A) Confirmation of Sp100A knockdown cells (Sp100Akd) by immunoblotting. (The Scr and Sp100Akd-1 and -2 cell lines were generated by lentivirus-mediated transduction of scramble shRNA and Sp100A shRNA1 and shRNA2.) (B) Confirmation of Flag-Sp100A expression in Sp100Aoe cells by immunoblotting with anti-Flag antibody. (C) Confirmation of Sp100Aoe cells by immunofluorescence staining with an anti-Flag antibody. (D and E) HEp-2 cells were mock infected or infected with VSV-GFP, PR8, DENV-2 (DENV), and ZIKV at an MOI of 5. At 6 hpi, whole-cell lysates were immunoblotted with antibodies reactive to Sp100 or β-actin (D), or cytosolic and nuclear lysates were loaded and immunoblotted with antibodies reactive to the indicated antibodies (E). (F) BEASE-2B cells were infected as in panel D, and the cytosolic and nuclear lysates were examined. (G) HEp-2 cells were transfected with plasmids encoding individual Flag-tagged Sp100 isoforms. Nuclear and cytosolic fractions were immunoblotted with anti-Flag antibody. (H) Cytosolic Sp100 expression was detected in various human cell lines by polyclonal anti-Sp100 antibody. The nuclear extract of HEp-2 cells served as a positive control (Con), and the loading volume of samples from each cell line was adjusted to avoid overexposure. (I) HEp-2 cells were mock infected or infected with VSV-GFP at an MOI of 0.1. At 24 hpi, the mRNA levels of IFN-α and Sp100 isoforms were quantified by qRT-PCR with Sp100 isoform-specific primers. (J) Aliquots of Flag-tagged NLS-expressing HEp-2 cells were seeded onto slides and then exposed to IFN-β. At the indicated time points post-IFN exposure, cells were fixed and subjected to immunofluorescence staining with Alexa 488-labeled NLS and Alexa 594-labeled NUP98. (K) Multiple cell lines were exposed to CHX only (NT) or CHX plus IFN-β (IFN-β) for 6 h, and the subcellular distribution of Sp100 was examined by immunoblotting using a polyclonal antibody against Sp100 (with 40 μL out of 600 μL total cytosolic lysate and 40 μL out of 200 μL total nuclear lysate loaded). (L) Samples from panel E were loaded on the same gel (with 30 μL out of 600 μL cytosolic lysate and 30 μL out of 200 μL nuclear lysate loaded), immunoblotted with polyclonal anti-Sp100 antibody, and exposed for the indicated time. Download FIG S1, TIF file, 2.2 MB. Copyright © 2022 Dong et al. 2022 Dong et al. https://creativecommons.org/licenses/by/4.0/ This content is distributed under the terms of the Creative Commons Attribution 4.0 International license.

To investigate whether the nuclear importation of Sp100A was an energy-dependent process, HEp-2 cells were deprived of ATP resources. As shown in Fig. 1J, ATP depletion blocked the nuclear translocation of Sp100A in response to IFN, suggesting that the process is an active transportation event. Furthermore, nuclear Sp100 could shuttle back to the cytosol, which was blocked by leptomycin B treatment (Fig. 1K). Overall, these observations indicate that Sp100A localizes in both the nucleus and cytosol and shuttles between these subcellular compartments and that IFN signaling promotes immediate enhancement of nuclear localization of Sp100A.

### Type I IFN increased the phosphorylation of cytosolic Sp100A on Ser^188^ through the PI3K pathway, fortified its binding affinity to PKM2, and promoted its nuclear importation.

**FIG 2:**
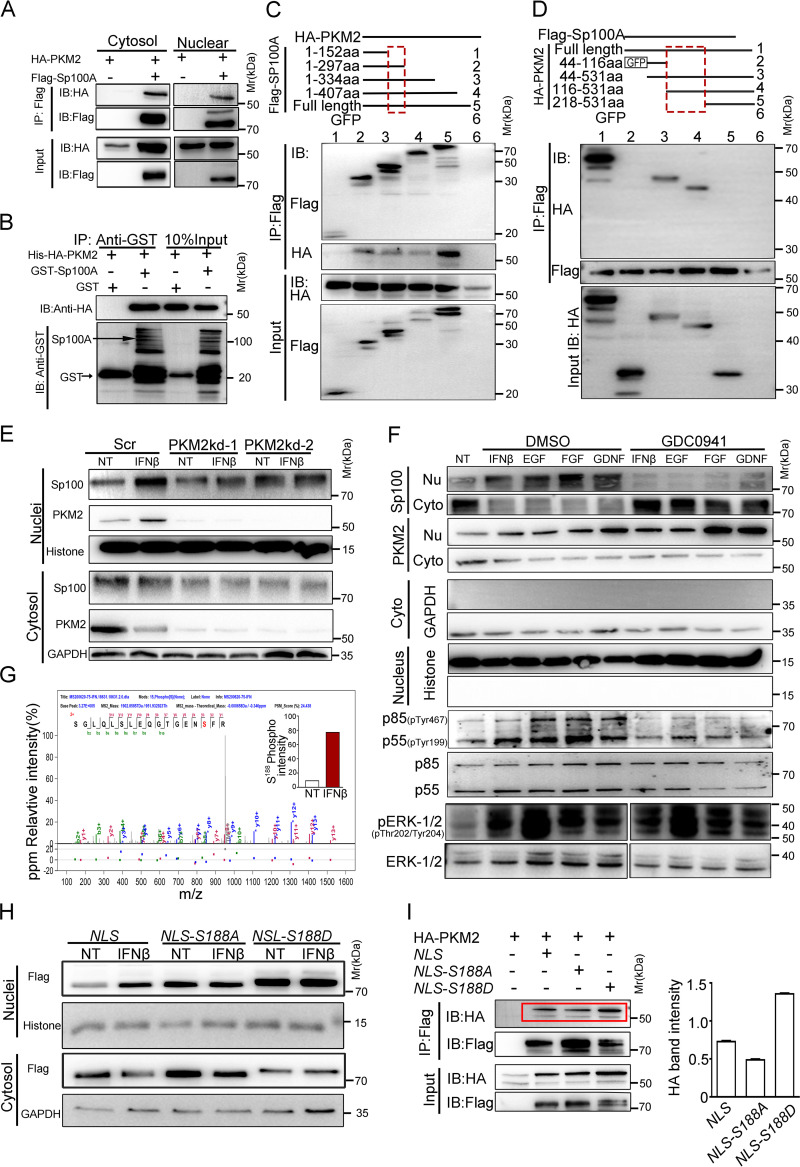
IFN increased the phosphorylation of Sp100A on Ser^188^ through the PI3K pathway, its binding affinity to PKM2, and subsequent nuclear importation. (A) Flag-Sp100A and HA-PKM2 were cotransfected into HEp-2 cells. Nuclear and cytosolic proteins were immunoprecipitated with anti-Flag antibody and then immunoblotted with anti-HA and anti-Flag antibodies. (B) Bacterially purified His-HA-PKM2 and GST-Sp100A were coincubated for 12 h at 4°C. Immunoprecipitation was performed with GST-Sepharose beads followed by immunoblotting with anti-GST or anti-HA antibodies. (C and D) Full-length or truncated Flag-Sp100A was cotransfected with HA-PKM2 (C), or full-length or truncated HA-PKM2 was cotransfected with Flag-Sp100A (D). Whole-cell lysates were immunoprecipitated with anti-Flag antibody and then immunoblotted with anti-HA and anti-Flag antibodies. (E) Cells stably expressing scramble shRNA (Scr) or shRNAs targeting PKM2 (PKM2kd-1 and PKM2kd-2) were treated with CHX only (NT) or IFN-β plus CHX (IFNβ) for 2 h. Nuclear and cytosolic proteins were immunoblotted with anti-Sp100 (Sp100 band exposure times of 10 s for the nuclear portion and 30 s for the cytosolic portion) and anti-PKM2 antibodies. (F) HEp-2 cells or GDC0941-pretreated HEp-2 cells were exposed to CHX only (NT) or CHX plus the indicated cytokines along with dimethyl sulfoxide (DMSO) or GDC0941 for 6 h. Nuclear and cytosolic lysates were immunoblotted with the indicated antibodies (Sp100 band exposure times of 3 s for the nuclear portion and 30 s for the cytosolic portion). (G) NLS protein was immunoprecipitated from the cytosol of nontreated or IFN-β treated (for 2 h) HEp-2 cells and sent for LC-MS/MS to identify protein modifications. The peptide with phosphorylated Ser^188^ was identified. The relative phosphorylation level of Sp100A at Ser^188^ in the NT group and IFN-β group was quantified by normalization of the peak area of the phosphorylated peptide to the peak area of the total peptide and plotted in the figure. (H) HEp-2 cells transfected with NLS or the NLS-S188A or NLS-S188D mutant were untreated or treated with IFN-β. Subcellular fractions were immunoblotted with anti-Flag antibody. (I) HA-PKM2 was cotransfected with GFP, NLS, or the NLS-S188A or NLS-S188D mutant in HEp-2 cells. Whole-cell lysates were immunoprecipitated with anti-Flag antibody, followed by immunoblotting with anti-HA and anti-Flag antibodies. The band density of HA-PKM2 immunoprecipitated by NLS or the NLS-S188A or NLS-S188D mutant (red rectangle) was quantified by ImageJ and is shown in the right panel.

Sp100A contains a functional nuclear localization signal (NLS) sequence and an identified SUMOylation site that are reported to be associated with nuclear importation of the protein (43, 44). Mutation at the NLS sequence or at both the NLS sequence and the Lys297 SUMO conjugation site (NLS/297) in Sp100A had no impact on the nuclear recruitment efficiency of the protein in response to IFN-β, although NLS mutation did lead to more retention of Sp100A in the cytoplasm (Fig. S2A and B). To facilitate investigation, NLS-mutated Sp100A was used in some experiments. To identify the potential interacting partner of Sp100A mediating its nuclear importation, cytosolic proteins bound by the transfected NLS mutant in IFN-β-treated HEp-2 cells or by bacterial purified glutathione *S*-transferase (GST)-tagged Sp100A during extracellular incubation with cytosolic proteins from IFN-β-treated HEp-2 cells were identified by mass spectrometry (MS). Eighteen proteins were identified in common under two experimental conditions (Fig. S2C), and pyruvate kinase 2 (PKM [accession no. P14618]) stood up as one of our candidates of interest due to its subcellular shuttling nature and nuclear translocation property upon epidermal growth factor receptor (EGFR) activation (Fig. S2D) (45, 46). Serial experiments were thus conducted to investigate the interaction between PKM2 and Sp100A. As shown in Fig. 2A, Sp100A coimmunoprecipitated with PKM2 in both cytosolic and nuclear compartments and increased the overall PKM2 protein level when overexpressed, and the two proteins shared overlapping intracellular localizations (Fig. S2E). To determine whether they directly interact with each other, bacterially purified GST-tagged Sp100A and His-tagged hemagglutinin (HA)-PKM2 were used for immunoprecipitation. GST-Sp100A, but not the GST tag, pulled down His-HA-PKM2 (Fig. 2B). Further analysis of the protein domains mediating the interaction between the two proteins showed that aa 152 to 297 of Sp100, containing a destruction box (d-box) (aa 165 to 168) (47), interacted with the B1 domain of PKM2 (aa 116 to 218) (48) (Fig. 2C and D).

10.1128/mbio.02044-22.3 FIG S2 Investigation of the molecular mechanisms regulating nuclear recruitment of Sp100A in response to IFN. (A and B) HEp-2 cells transfected with plasmids expressing NLS or NLS/297 for 24 h were untreated (NT) or treated with IFN-β for 6 h. (A) Nuclear and cytoplasmic fractions were immunoblotted with the indicated antibodies. (B) Cells were fixed and subjected to immunofluorescence staining with anti-Flag antibody labeled with Alexa 488 and anti-PML antibody labeled with Alexa 594. (C) Cytosolic proteins in IFN-β-treated HEp-2 cells bound by transfected NLS or by bacterial purified GST-Sp100A during extracellular incubation were identified by mass spectrometry. A Venn diagram shows the cytosolic proteins identified by both methods. (D) Tandem mass spectra identified the unique peptide of PKM2 that precipitated with GST-Sp100A in panel C. (E) HEp-2 cells were transfected with GFP and HA-PKM2 or Flag-Sp100A and HA-PKM2. At 24 h posttransfection, cells were fixed with 4% PFA and stained with Alexa 488-labeled anti-Flag antibody and Alexa 594-labeled anti-HA antibody. Alexa 594-labeled GAPDH served as a control. (F) HEp-2 cells transfected with scramble siRNA (siScr) or PIN1 siRNA were treated with CHX only (NT) or CHX plus IFN-β (IFNβ) for 2 h, and subcellular fractions were immunoblotted with the indicated antibodies. (G) HEp-2 cells transfected with scramble siRNA (siScr) or importin-α or importin-β siRNA were treated with CHX only or CHX plus IFN-β for 2 h. Subcellular fractions were immunoblotted with anti-Sp100 antibody (SP100 band exposure times of 3 s for the nuclear portion and 30 s for the cytosolic portion in panels F and G). (H) The predicted crystal structures of SP100A and PKM2 were downloaded from the AlphaFold Protein Structure Database. The interaction model with the highest HADDOCK score by HADDOCK 2.4 is shown. Download FIG S2, TIF file, 2.1 MB. Copyright © 2022 Dong et al. 2022 Dong et al. https://creativecommons.org/licenses/by/4.0/ This content is distributed under the terms of the Creative Commons Attribution 4.0 International license.

Previous studies imply that PKM2 is activated by ERK1/2 upon EGF signaling and transported into the nucleus by interacting with PIN1 and importin α (46). To investigate the role of PKM2 in the nuclear transportation of Sp100A in response to IFN, two PKM2 knockdown cell lines (PKM2kd-1 and PKM2kd-2) and a control scramble short hairpin RNA (shRNA)-transduced cell line (Scr) were established. As shown in Fig. 2E, PKM2 translocated into the nucleus upon IFN-β treatment in Scr cells, as did Sp100A. In comparison, neither an obvious increase in nuclear Sp100A nor a distinguishable reduction in cytosolic Sp100A was observed in PKM2kd cells, implying the reliance of IFN-signaled Sp100A nuclear recruitment on the presence of PKM2. In addition, interference with the protein expression of PIN1 or importins by small interfering RNA (siRNA) significantly blocked the nuclear importation of PKM2 and Sp100 upon IFN stimulation (Fig. S2F and G), implying that Sp100A utilizes the ERK1/2-PKM2-PIN1-importin axis for its own nuclear importation in response to IFN.

To further understand the signaling cascade regulating the subcellular relocation of Sp100A in response to IFN, multiple inhibitors targeting various signaling pathways potentially activated by type I IFN were investigated. The inhibitor GDC0941, which blocks the PI3K pathway, significantly reduced the phosphorylation level of PI3K p85/p55 and abolished the nuclear importation of Sp100A in response to IFN but did not affect the phosphorylation level of ERK1/2 or the nuclear translocation of PKM2 (Fig. 2F). These results suggest that IFN regulates the relocation of cytosolic Sp100A through the PI3K pathway. In efforts to identify the molecular modifications on Sp100A during this process, Sp100A from the cytosolic compartment of nontreated (NT) and IFN (IFN-β)-treated HEp-2 cells was sent for liquid chromatography-tandem mass spectrometry (LC-MS/MS). A total of 8 phosphorylation sites on Sp100A were identified: 5 appeared in both groups, and 3 were unique to either the NT or IFN-β group (Table S1). The phosphopeptide containing Ser^188^ was identified in tandem mass spectra, whose ion count in the IFN-β group was significantly higher than that in the NT group (Fig. 2G). Importantly, substitution of Ser^188^ with alanine in the NLS (NLS-S188A) led to severe cytosolic retention, while phosphor-mimicking mutation of Ser^188^ to aspartic acid in the NLS (NLS-S188D) induced an overall increase in the nuclear versus cytosolic levels of the protein. Both mutants failed to translocate into the nucleus in response to IFN-β, suggesting that phosphor-Ser^188^ of Sp100A was critical for IFN-β signaled nuclear relocation of the protein (Fig. 2H). As Ser^188^ of Sp100A is located within the interaction domain of Sp100A with PKM2 (Fig. 2C and Fig. S2H), the role of phospho-Ser^188^ of Sp100A in this protein-protein interaction was examined. HA-PKM2 was cotransfected with the Flag-tagged NLS or the NLS-S188A or NLS-S188D mutant, and the band intensity of HA-PKM2 coimmunoprecipitated down by NLS or NLS mutants was quantified. While NLS-S188D exhibited the highest affinity to PKM2, substitution of S188 with A in Sp100A decreased the protein’s binding tendency to PKM2 (Fig. 2I). The data implies that phospho-Ser^188^ of Sp100A facilitates the relocation of the protein by strengthening its affiliation with PKM2 in the ERK1/2-PKM2-PIN1-importin axes of nuclear importation.

10.1128/mbio.02044-22.6 TABLE S1 Mass spectrometry identified phosphorylation sites on cytosolic Sp100A. Mass spectrometry identified phosphorylation sites on cytosolic Sp100A in both the untreated and IFN-β-treated groups, and these potential modification sites were compared and named to facilitate labeling. Download Table S1, DOCX file, 0.02 MB. Copyright © 2022 Dong et al. 2022 Dong et al. https://creativecommons.org/licenses/by/4.0/ This content is distributed under the terms of the Creative Commons Attribution 4.0 International license.

### IFN-β signaling led to enrichment of Sp100A in the promoter region of multiple antiviral ISGs and promoted their transcription.

**FIG 3:**
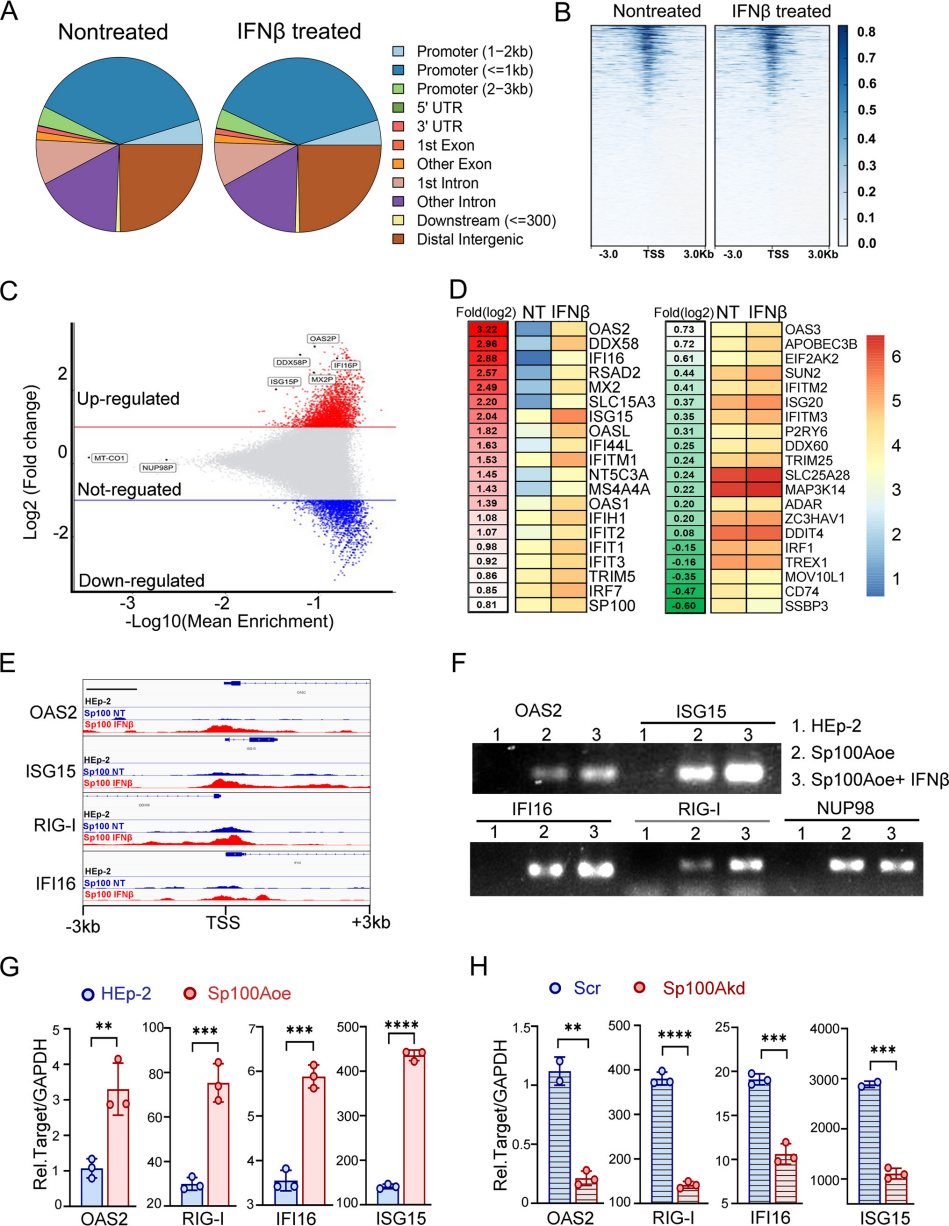
Sp100A was assembled in the promoter region of antiviral ISGs in response to IFN-β and positively correlated with their transcription. (A) Chromosomal regions bound by Sp100A in nontreated (NT) and IFN-β-treated (IFNβ) HEp-2 cells; (B) heat maps showing Sp100A CUT&Tag normalized read counts at TSSs for the NT and IFN-β groups. The signal was displayed from −3 kb to +3 kb surrounding each annotated TSS. Gene promoters were ordered by read counts. (C) MA plot showing alterations in the Sp100A association frequency in the promoter regions of cellular genes in response to IFN-β. Horizontal red and blue lines define −log_2_ fold changes of >1 and <−1, respectively. (D) Relative enrichment of Sp100A on the promoter regions of 40 antiviral ISGs. The exact enrichment level of Sp100A on the promoter of each gene in the IFN-β group normalized to the NT group is listed to the left of the genes’ products as fold (log_2_) change. (E) Chromatin landscape across the promoter region of OAS2, RIG-I, IFI16, and ISG15 for Sp100A CUT&Tag in NT and IFN-β-treated HEp-2 cells. (F) The promoter region of the genes coding for the indicated proteins was amplified from Sp100A CUT&Tag libraries of HEp-2 cells (lane 1), the Sp100oe NT group (lane 2), and the Sp100oe IFN-β group (lane 3) with limited cycles of PCR (primer infromation in Table S3) and then run on the gel. (G and H) mRNA levels of the indicated genes coding for the proteins shown were measured by qPCR in HEp-2 and Sp100Aoe cells (G) and in HEp-2 cells expressing scramble shRNA (Scr) and Sp100Akd cells (H). The two-tailed unpaired Student's *t* test was used to calculate *P* values in panels G and H.

Sp100 protein has been previously reported to contain direct DNA binding domains or to interact with chromosomal proteins to regulate the transcription of genes of both cellular and viral origins (33, 43, 49–55). Thus, investigation of how IFN-β shifts the chromosomal association pattern of Sp100A may help to elucidate the biological function of this isoform in response to IFN. In this study, the CUT&Tag (cleavage under targets and tagmentation) technique plus Illumina deep sequencing (deep-seq) were employed to analyze Sp100A-associated host chromosomal regions in NT and IFN-β-treated Sp100Aoe cells (56). The sample from HEp-2 cells served as a negative control because it lacked Flag-Sp100A expression. General chromosome association pattern profiling of Sp100A showed that the protein was preferentially associated with the promoter region within a −3-kb to +3-kb distance from the transcription start site (TSS) and was most frequently located within the transcription regulation area less than 1 kb from the TSS, reinforcing its major role as a transcriptional regulator. (Fig. 3A and B). Further in-depth comparison of the enrichment level of Sp100A in the promoter regions between the NT and IFN-β groups revealed the following findings. (i) IFN stimulation substantially changed the promoter region binding affinity of Sp100A on a large group of targeted genes. Among the promoters with increased Sp100A association frequency in response to IFN-β, the top hits included promoters of ISGs such as OAS2P, DDX58P, IFI16P, MX2P, and ISG15P (Fig. 3C). (ii) Comparison of the enrichment degree of Sp100A in the promoter regions of a set of experimentally confirmed antiviral ISGs, reviewed by Charles Rice in 2011, revealed an overall enrichment of Sp100A on these ISGs upon IFN signaling (57, 58) (Fig. 3D). Specifically, Sp100A was preferentially assembled on the promoters of OAS2, RIG-I (DDX58), IFI16, RSAD2 (Viperin), MX2, SLC15A3, and ISG15, with more than 5-fold-higher enrichment in the IFN-β group than in the NT group (Fig. 3D and E). To experimentally validate the CUT&Tag result, primers flanking the promoter region of OAS2, ISG15, RIG-I, IFI16, and NUP98 (negative control) (Fig. 3C) were used for limited-cycle PCR to semiquantify their abundance in the CUT&Tag libraries from HEp-2 cells, NT Sp100Aoe cells, or IFN-β-treated Sp100Aoe cells. Consistent with the deep-seq data, samples from HEp-2 cells gave no positive amplification results for any promoter regions, and IFN-β treatment of Sp100oe cells led to increased association of Sp100A with the promoter regions of OAS2, ISG15, DDX58, and IFI16 but not NUP98 (Fig. 3F).

The biological consequence of the increased association of Sp100A with the promoter region of the ISGs was investigated by quantification of the transcription levels of OAS2, ISG15, RIG-I, and IFI16 in HEp-2, Sp100Aoe, and Sp100Akd cells, and the results showed that the transcription of these genes positively correlated with the cellular abundance of the Sp100A protein (Fig. 3G and H).

### Nuclear translocation of Sp100A promoted the expression of antiviral ISGs during virus infection and restricted replication of VSV *in vitro*.

**FIG 4:**
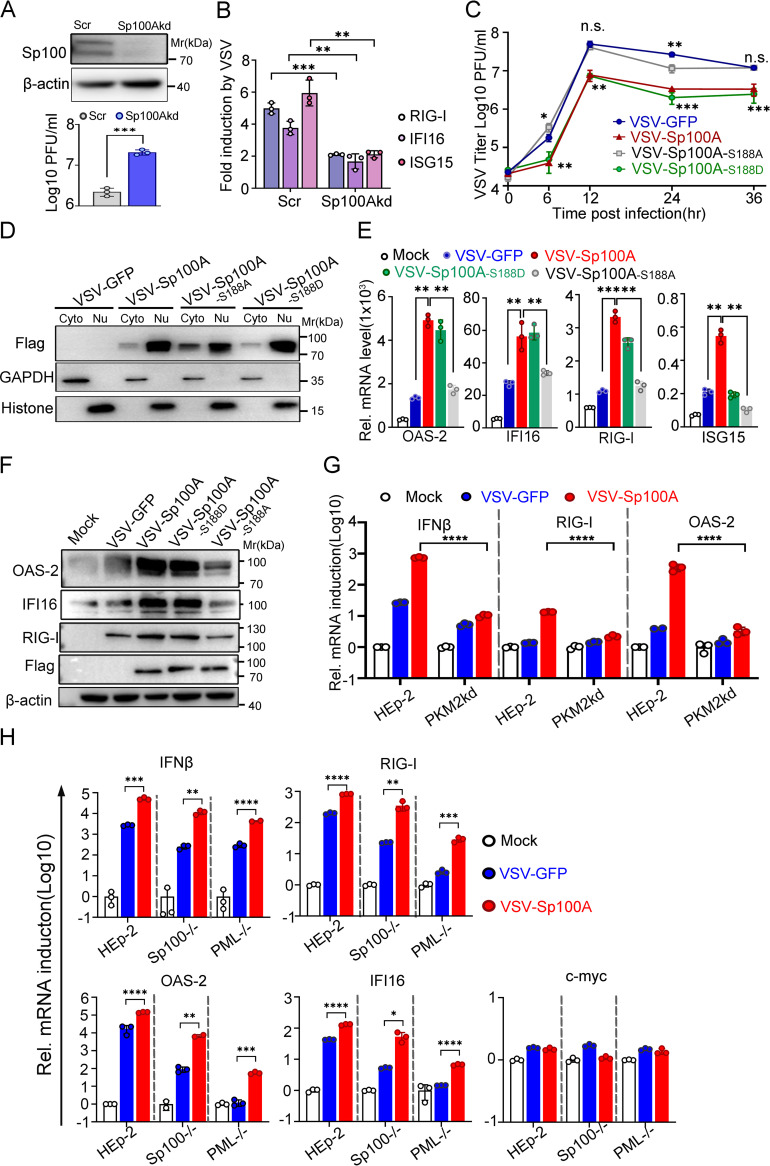
Nuclear translocation of Sp100A mediated the innate immune response against VSV and restricted viral replication *in vitro*. (A) Confirmation of Sp100A knockdown in BEASE-2B cells by immunoblotting (upper panel). Sp100Akd and Scr cells were infected at an MOI of 0.1 for 24 h, and virus yield was titrated by plaque assay (lower panel). (B) Sp100Akd and Scr cells were infected at an MOI of 0.1 for 6 h, and the mRNA levels of RIG-I, IFI16, and OAS2 were quantified by qRT-PCR. (C) Growth kinetics of VSV-GFP, VSV-Sp100A, VSV-Sp100A-S188D, and VSV-Sp100A-S188A in HFL1 cells at an MOI of 1. Statistical differences in the virus titers of VSV-Sp100A-S188A and VSV-Sp100A-S188D compared to VSV-GFP at each time point are shown. (D) HFL1 cells were infected as in panel C at an MOI of 5. Cells were collected at 6 hpi, and the subcellular distributions of Flag-Sp100A, Flag-Sp100A S188A, and Flag-Sp100A S188D were examined by immunoblotting (with cytosolic and nuclear lysates loaded proportionally in the panel). (E) HFL1 cells were infected at an MOI of 0.1 for 6 h (12 h for the detection of IFI16). The expression levels of OAS2, IFI16, RIG-I, and ISG15 were quantified by qRT-PCR. (F) HFL1 cells were infected with the indicated viruses at an MOI of 0.1 for 24 h. The protein levels of OAS2, RIGI, IFI16, and Flag-Sp100A were examined by immunoblotting. (G and H) HEp-2 and PKM2kd cells (G) or HEp-2, PML^−/−^, and Sp100^−/−^ cells (H) were mock infected or infected with the indicated viruses at an MOI of 1 for 6 h. The mRNA levels of the indicated gene products in panels G and H were quantified by qRT-PCR. Two-tailed unpaired Student's *t* test was used to calculate *P* values.

The biological function of Sp100A as a host restrictive factor during infection was first reconfirmed in a nontumorigenic cell line. Knockdown of Sp100A promoted VSV replication efficiency in BEAS-2B cells (Fig. 4A) and significantly reduced the transcriptional activation of RIG-I, IFI16, and ISG15 (Fig. 4B).

To ascribe this biological consequence to the Ser^188^ phosphorylation of Sp100A during virus infection, recombinant VSV expressing GFP (VSV-GFP), Sp100A (VSV-Sp100A), an Sp100A-S188A mutant (VSV-Sp100A-S188A), or an Sp100A-S188D mutant (VSV-Sp100A-S188D) was constructed and rescued (Fig. S3A to C). While expression of wild-type Sp100A or Sp100A-S188D significantly inhibited VSV replication, recombinant VSV carrying the Sp100A-S188A mutant grew similarly to the recombinant VSV inserted with GFP in multiple cell lines (Fig. 4C and Fig. S3D and E). The virulence of recombinant VSV strains was negatively correlated with the nuclear translocation capacity of their carried Sp100A mutants during infection (Fig. 4D and Fig. S3F). To examine the expression level of the previously identified Sp100A-regulated ISGs, HFL1 cells or HEp-2 cells were mock infected or infected with VSV-GFP, VSV-Sp100A, VSV-Sp100A-S188D or VSV-Sp100A-S188A. The results revealed that the transcription levels of IFN-β, OAS2, RIG-I, ISG15, and IFI16 were significantly higher in VSV-Sp100A- and VSV-Sp100A-S188D-infected cells than in cells inoculated with VSV-GFP and that replacement of Sp100A with Sp100A-S188A in recombinant VSV remarkably reduced the protein’s capability to induce the above ISGs during virus infection (Fig. 4E and Fig. S3G). Consistent with their pattern of transcription, the protein levels of OAS2, RIG-I, and IFI16 were significantly upregulated in HFL1 cells infected with VSV-Sp100A compared with HFL1 cells infected with VSV-GFP or VSV-Sp100A-S188A (Fig. 4F and Fig. S3H). We thus conclude that Ser^188^ phosphorylation of Sp100A is positively associated with its nuclear translocation capacity, its transcriptional activation of essential antiviral ISGs, and its antiviral potency during VSV infection. Of note, VSV-Sp100A-S188D behaved similarly to VSV-Sp100A in the tests performed in this report, and we argue that this is a plateau effect.

10.1128/mbio.02044-22.4 FIG S3 Investigation of the role of the nuclear translocation of Sp100A during VSV infection. (A) Expression of Flag-Sp100A in VSV-Sp100A-infected HEp-2 cells at an MOI of 1 at 24 hpi by immunoblotting. (B and C) HEp-2 cells were infected with the indicated viruses at an MOI of 5. At 6 hpi, cells were fixed and stained with Alexa 488-labeled anti-PML antibody and Alexa 594-labeled anti-Flag antibody (B) or stained with Alexa 594-labeled anti-Flag antibody (C). (D) HEp-2 cells were infected at an MOI of 1 and imaged at 24 hpi. (E) Growth kinetics of VSV-GFP, VSV-Sp100A, VSV-Sp100A-S188D, and VSV-Sp100A-S188A in BEAS-2B and HEp-2 cells at an MOI of 1. (F) HEp-2 cells were infected at an MOI of 5. At 6 hpi, the subcellular distribution of Flag-Sp100A, VSV-Sp100A-S188A and Flag-Sp100A S188D was examined by immunoblotting (with cytosolic and nuclear lysates loaded proportionally in the panel). HEp-2 cells were infected at an MOI of 0.1 (G) or MOI of 1 (H). At 6 hpi, the mRNA levels of OAS2, RIGI, and IFI16 were measured by qRT-PCR (G). At 24 hpi, the protein levels of OAS2, RIGI, IFI16, and Flag-Sp100A were examined by immunoblotting (H). (I) IFI16, RIG-I, and ISG15 mRNA expression in Sp100^−/−^ cells transfected with red fluorescent protein (RFP)- or HA-PKM2-expressing plasmids were quantified by qPCR. J. HEp-2, or Sp100Aoe cells transfected with Scr siRNA or PKM2 siRNA for 24 h were infected with VSV-GFP at an MOI of 1, the samples were collected at 12 and 24 hpi, and the virus titer was determined. (K) HEp-2 or its derived cell lines as indicated were infected at an MOI of 0.1, and at 12 hpi, the IFN-β concentration in the culture medium was quantified by ELISA. Two-tailed unpaired Student’s *t* test was used to calculate *P* values in panels G, I, J, and K. Download FIG S3, TIF file, 2.2 MB. Copyright © 2022 Dong et al. 2022 Dong et al. https://creativecommons.org/licenses/by/4.0/ This content is distributed under the terms of the Creative Commons Attribution 4.0 International license.

Previously, in this study, we identified PKM2 as an essential partner for nuclear importation of Sp100A. We further confirmed that PKM2 itself had no promotive role in activating ISGs (Fig. S3I). A logical inference is that targeting PKM2 should diminish Sp100A nuclear translocation during VSV infection and weaken its transcriptional activation of OAS2, RIG-I, and IFI16. Hence, PKM2kd cells or HEp-2 cells were mock infected or infected with VSV-GFP or VSV-Sp100A, and the expression levels of OAS2, RIG-I, and IFN-β during infection were quantified by quantitative PCR (qPCR). The transcription levels of OAS2, RIG-I, and IFN-β during VSV-Sp100A infection were significantly reduced in PKM2kd cells compared to HEp-2 cells (Fig. 4G). Knockdown of PKM2 in Sp100Aoe cells alleviated the inhibitory effect on virus growth imposed by Sp100A overexpression (Fig. S3J).

A scientifically important inquiry is the dependency of the function of Sp100A, investigated in this study, on intact PML bodies and other Sp100 isoforms (10, 11). VSV-Sp100A infection in PML^−/−^ and Sp100^−/−^ cells induced significantly higher levels of transcription from RIG-I, OAS-2, IFI16, and IFN-β genes than VSV-GFP did but not transcription from the control gene c-*myc* (Fig. 4H). It should be noted that depletion of PML protein drastically impaired the overall transcriptional induction of RIG-I, OAS2, and IFI16 during VSV infection (Fig. 4H) (5, 7–10, 33, 59, 60). Secreted IFN-β from VSV-GFP- and VSV-Sp100A-infected HEp-2, Sp100^−/−^, PML^−/−^, and PKM2kd cells was quantified by enzyme-linked immunosorbent assay (ELISA), and the protein levels of IFN-β agreed with its transcriptional pattern (Fig. S3K).

### Nuclear translocation of Sp100A restricted replication of VSV *in vivo*.

**FIG 5:**
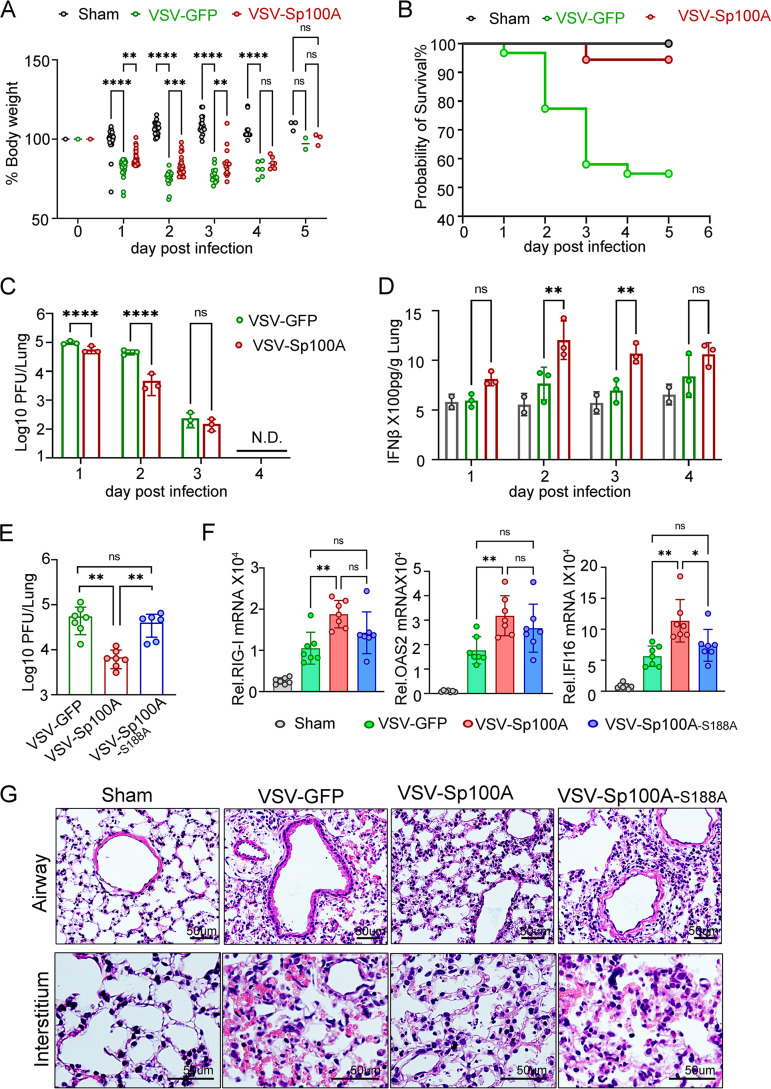
The growth of VSV was inhibited by Sp100A carried by VSV *in vivo* but not much by Sp100A-S188A. (A to D) BALB/c mice were sham infected with DMEM only (*n* = 31) or infected with VSV-GFP (*n* = 31) or VSV-Sp100A (*n* = 31) as described in Materials and Methods. (A) Body weight of mice from different groups was measured every day, normalized to their original weight at 0 dpi, and plotted. (B) Survival curve of the infected mice. (C) At the indicated days postinfection, the virus titer in infected mouse lungs was measured by plaque assay. (D) The IFN-β concentration in lung homogenates from each group was measured by ELISA, and the final concentration was normalized to organ weight and plotted. (E to G) BALB/c mice were inoculated with VSV-GFP (*n* = 15), VSV-Sp100A (*n* = 15), VSV-Sp100A-S188A, or sham infected with DMEM (*n* = 15). (E) At day 2 postinfection, the virus titer in the lung was measured. (F) mRNA expression levels of OAS2, RIGI, and IFI16 in the lungs were quantified by qRT-PCR. (G) At day 3 postinfection, lung sections of mice inoculated with VSV-GFP, VSV-Sp100A, or VSV-Sp100A-S188A or sham infected with DMEM were sent for H&E staining. To calculate *P* values, two-way ANOVA was used in panels A, C, D, and F and two-tailed unpaired Student's *t* test was used in panel E.

Despite species-specific sequence differences, Sp100A inhibited VSV replication in the murine cell line L929, implying functional conservation of human Sp100A in murine cells (Fig. S4A). Thus, to investigate the function of Sp100A *in vivo*, we compared the pathogenesis of VSV-GFP and VSV-Sp100A in BALB/c mice infected through the intranasal inoculation route. In general, all recombinant VSV strains showed limited replication in mouse lungs during the first few days, leading to loss of body weight, the experimental endpoint when the humane endpoint of weight loss was reached, or mortality of the infected animals. As shown in Fig. 5A and B, VSV-Sp100A-infected mice showed significantly less weight loss than VSV-GPF-infected mice during the first 3 days postinfection and had a higher survival rate. Although viruses were cleared in the lung tissue of surviving mice on day 4 in both the VSV-GFP- and VSV-Sp100A-infected groups, VSV-GFP replicated to a significantly higher titer in the lungs at day 1 and day 2 postinfection than VSV-Sp100A (Fig. 5C) and induced less IFN-β production in the lung homogenate fluid collected at days 2 and 3 postinfection (Fig. 5D).

10.1128/mbio.02044-22.5 FIG S4 VSV-Sp100A was attenuated in mice. (A) Single-cycle growth kinetics of VSV-GFP, VSV-Sp100A, and VSV-Sp100A-S188A in L929 cells (MOI = 5), titrated by plaque assay in Vero cells. (B and C) BALB/c mice were inoculated with VSV-GFP, VSV-Sp100A, or VSV-Sp100A-S188A or sham infected with DMEM. (B) At day 2 postinfection, total RNA was extracted from mouse lungs using TRIzol reagent, and the relative viral genome level in the lungs was quantified by qRT-PCR. C. At day 3 postinfection, mice were sacrificed, and lungs were fixed via cardiac perfusion as described in the supplemental materials and methods in Text S1. Representative images of 4% PFA-perfused lungs are shown. (D) At day 3 postinfection, lung sections of mice inoculated with VSV-GFP, VSV-Sp100A, or VSV-Sp100A-S188A or sham infected with DMEM were sent for H&E staining and imaged under various magnifications. Two-tailed unpaired Student’s *t* test was used in panel B to calculate *P* values. Download FIG S4, TIF file, 2.6 MB. Copyright © 2022 Dong et al. 2022 Dong et al. https://creativecommons.org/licenses/by/4.0/ This content is distributed under the terms of the Creative Commons Attribution 4.0 International license.

10.1128/mbio.02044-22.1 TEXT S1 Experimental details of the reagents and methods used. Download Text S1, DOCX file, 0.02 MB. Copyright © 2022 Dong et al. 2022 Dong et al. https://creativecommons.org/licenses/by/4.0/ This content is distributed under the terms of the Creative Commons Attribution 4.0 International license.

To compare the antiviral capabilities of Sp100A and the Sp100A-S188A mutant *in vivo*, BALB/c mice were inoculated intranasally with VSV-GFP, VSV-Sp100A, or VSV-Sp100A-S188A or sham infected with phosphate-buffered saline (PBS). On day 2 postinfection, infected animals were sacrificed, and the virus titer in the infected lungs as well as the total mRNA level of the Sp100A-regulated ISGs in lung tissue were quantified. As shown in Fig. 5E and Fig. S4B, replacement of Ser^188^ with an alanine in Sp100A partially restored the replication capacity of the recombinant VSV in mice. Furthermore, VSV-Sp100A-S188A was not distinguishable from VSV-GFP regarding the capability to induce RIG-I, IFI16, and OAS2 in mice, while VSV-Sp100A induced significantly higher levels of RIG-I, OAS2, and IFI16 than VSV-GFP (Fig. 5F). Lungs from differentially infected mouse groups collected at day 3 postinfection were fixed through perfusion (Fig. S4C) and sent for hematoxylin and eosin (H&E) staining. While VSV-GFP and VSV-Sp100A-S188A infection caused significant interstitial infiltration, evident airway epithelium thickening, edema, and changes in the alveolar structures in the lungs of infected animals, VSV-Sp100A-infected lung tissue displayed better alveolar structures and no apparent epithelium thickening, implying much milder overall pathogenic damages (Fig. 5G and Fig. S4D).

## DISCUSSION

PML bodies play an essential role in mounting antiviral status in response to IFN. However, little is known about the molecular mechanisms mediating signal transduction from IFN signaling to PML bodies and the subsequent regulation of cellular responses (61). Previously, our group reported that the PML body component Sp100A is actively exported to and mediates antiviral responses in neighboring uninfected cells through extracellular vesicles during HSV-1 infection (42). In this study, we found that Sp100A is in both the nucleus and the cytoplasm of various types of human cells and that it shuttles between the two compartments through ATP-dependent nuclear importation and CRM1 (chromosomal region maintenance)/exportin 1-dependent nuclear exportation in multiple human cell lines of cancer and normal cell origins. Upon the addition of IFN-β, a portion of Sp100A in the cytoplasm was translocated into the nucleus within minutes, implying that the phenomenon was a primary response to IFN signaling. Note that we constantly observed a small portion retained in the cytosol and that cytosolic Sp100 did not show nuclear transportation in response to IFN in HEpG-2 cells (see Fig. S1K in the supplemental material). Both observations suggested that cytosolic Sp100A may respond to multiple different signals in the cytosol in a cell-type- and cell status-dependent manner.

Next, we identified that phosphorylation of Ser^188^ and activation of the PI3K pathway regulated the IFN-mediated nuclear recruitment of Sp100A through the ERK1/2-PKM2-PIN1-importin axes, and we provided evidence supporting the correlation among phosphorylation at Ser^188^ of Sp100A, Sp100A interaction with PKM2, and the nuclear importation of Sp100A in response to IFN. Note that although PKM2 interacted with Sp100A in both the nuclear and cytosolic compartments, PKM2 did not form aggregated puncta in the nucleus with Sp100A (Fig. S2E). It is important to investigate in the future whether PKM2 serves only as a shuttling vector for cytosolic Sp100A and the possible biological impact of this interaction on cellular metabolism.

The third inquiry in this study was the functionality of nuclear imported Sp100A in response to IFN. Although PML bodies and the Sp100 protein family have been extensively reported to participate in the transcriptional regulation of both host and viral genes, no precise linkage of Sp100A and ISGs has been described. The appealing findings of this study revealed that IFN stimulation led to significant enrichment of Sp100A on only a set of essential antiviral ISGs, including those coding for RIG-I, IFI16, MX2, OAS2, and ISG15. In the absence of a direct DNA binding domain, enrichment of Sp100A in the promoter regions seemed to significantly activate their transcription, independent of other Sp100 isoforms and PML protein/PML bodies, which raised two intriguing questions. First, we wondered whether the scaffold protein PML or PML bodies served merely as an organizer that facilitated biochemical interactions within the structure. Second, what is the molecular mechanism that mediates the enrichment of Sp100A in the promoter regions of the targeted ISGs? Nevertheless, the results reported here strongly indicate that the nuclear translocation of Sp100A is a primary response to IFN stimulation and/or virus infection to activate the antiviral status in cells.

**FIG 6:**
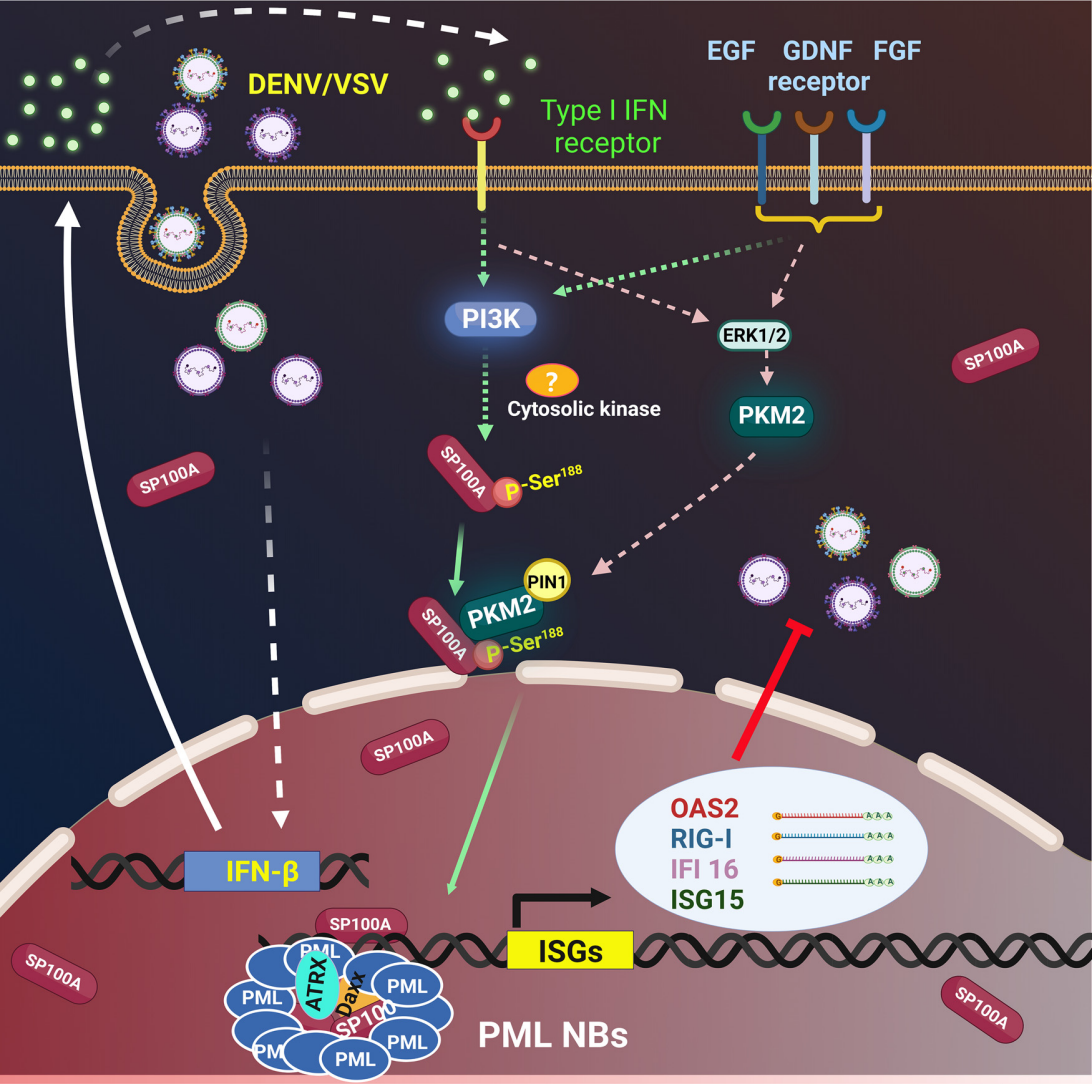
Proposed model of Sp100A activated by IFN signaling in the cytosol and actuating antiviral responses in the nucleus.

In summary, the findings in this study depict a novel IFN response mechanism by the PML body in the cytosol through its permanent component Sp100A. A proposed model is shown in Fig. 6. Note that the subcellular relocalization of Sp100A could be triggered by a variety of cellular stimuli, such as EGF, glial cell-derived nerve factor (GDNF), etc. (Fig. 2F). The subsequent intriguing questions involve whether cytosolic Sp100A, under such conditions, is differentially modified and/or interacts with different partner proteins for nuclear importation and further whether these changes reflect a general strategy of how PML bodies process massive and diverse cellular signals and “translate” them into transcriptional responses.

## MATERIALS AND METHODS

### Cell lines and viruses.

HEp-2, HEK293T, MCF-7, SH-SY5Y, BHK-21, BEAS-2B, MDCK, and HepG2 cells were maintained in Dulbecco’s modified Eagle’s medium (DMEM) (Corning; 10013074) supplemented with 10% fetal bovine serum (FBS). HFL, A549 and AKATA cells were maintained in RPMI 1640 basic medium (Gibco; 8121248) supplemented with 10% FBS. The Sp100A overexpression cell line (Sp100Aoe) and Sp100A isoform-specific knockdown cell line (Sp100Akd), IFNAR knockdown cell lines (IFNARkd) and PKM2 knockdown cell lines (PKM2kd-1, PKM2kd-2) were generated using a lentiviral system on the background of HEp-2 cells and BEAS-2B cells. A549-GFP and A549-SP100A cells were generated using a lentiviral system on the background of A549 cells. The Sp100 knockout cell line (Sp100^−/−^) and PML knockout cell line (PML^−/−^) were established using CRISPR/Cas9 in our lab (16, 17). Recombinant VSV viruses were rescued in BHK-21 cells and amplified in Vero cells as previously described (59). In brief, to construct recombinant VSV expressing GFP, Sp100A, Sp100A-S188A, or Sp100A-S188D, the indicated open reading frame (ORF) was amplified and inserted between the XhoI and NheI sites in the pVSV-XN2 vector. BHK-21 cells seeded in 6-well plates were infected with vVTF-7 at a multiplicity of infection (MOI) of 0.1 and then transfected with pVSV-XN2, PBS-N, PBS-P, and PBS-L (5:5:4:1 = 2 μg, 2 μg, 1.6 μg, and 0.4 μg, respectively). At 6 h posttransfection, the culture medium was replaced with DMEM containing 10% FBS. At 48 to 72 h post-vVTF-7 infection, the culture medium was collected and passed through a 0.2-μm-pore filter, and the rescued viruses in the medium were further amplified and plaque purified in Vero cells and stored at −80°C. Dengue virus subtype 2 (DENV-2) and Zika virus (ZIKV) were kindly provided by Zhongyu Liu and amplified and titrated in BHK-21 cells. Influenza virus strain PR8 was kindly provided by Yaoqing Chen and amplified and titrated in MDCK cells. The VSV recombination system was a kind gift from Deyin Guo. Mycoplasma routinely tested negative to ensure mycoplasma-free conditions throughout the study.

### Plasmids and transfection.

Flag-tagged human Sp100A and HA-tagged human PKM2 were cloned into the pcDNA3.1 or PLVX vector. Mutants or truncations of Sp100A were constructed by overlapping PCR using the primers in Table S2 and subcloned into the HindIII/NotI sites of a modified pcDNA3.1. All constructs were verified by sequencing. Transient transfection of plasmids into HEK293T cells was performed using standard polyethyleneimine (PEI) (Sigma; 408727). For HEp-2 cells and other mammalian cell lines, Jetprime (Polyplus; 101000046) was used for transfection of plasmids and siRNA according to the manufacturer’s protocol. Plasmids were transfected for 24 h, and siRNAs were transfected for 36 h before further experiments unless otherwise stated.

10.1128/mbio.02044-22.7 TABLE S2 Primers for plasmid and mutation construction. Download Table S2, DOCX file, 0.01 MB. Copyright © 2022 Dong et al. 2022 Dong et al. https://creativecommons.org/licenses/by/4.0/ This content is distributed under the terms of the Creative Commons Attribution 4.0 International license.

### Lentiviral transduction.

HEK293T cells were seeded in 60-mm dishes and transfected with the packaging plasmids VSV-G and Δ8.9 with PLVX-Flag-Sp100A, PLKO-shSp100A, or control vectors using PEI at the appropriate ratio. At 48 h posttransfection, culturing medium containing lentiviruses was collected and passed through a 0.45-μm-pore filter. HEp-2 cells were inoculated with lentiviruses for 4 h. At 48 h postinoculation (hpi), the cells were selected with hygromycin B (200 μg/mL) (Sigma; V900372) or puromycin (1 μg/mL) (Gibco; A1113803) for 4 to 7 days.

### Immunoprecipitation and mass spectrometry.

For immunoprecipitation of cytosolic Sp100A, cytosolic fractions of 1 × 10^7^ HEp-2 cells transfected with Flag-Sp100A NLS mutant or GFP were extracted. The cytosolic fraction was then incubated with anti-Flag antibody-conjugated magnetic beads overnight at 4°C. The precipitates were washed with PBST (PBS with 1% Triton X-100) 3 times, denatured at 95°C for 10 min and loaded onto polyacrylamide gels for Coomassie blue staining (staining reagent, 50% MeOH, 10% HoAC, 40% H_2_O, 0.25% Coomassie blue R-250; staining solution, 5% MeOH, 7.5% HoAC, 87.5% H_2_O) or for immunoblot analysis. Specific bands of the NLS mutant- and potential Sp100A-interacting proteins were cut and sent for liquid chromatography with tandem mass spectrometry (LC-MS/MS) at the National Protein Science Facility, School of Life Science, Tsinghua University, People’s Republic of China. The raw data analysis was performed using Pfind software (60, 61).

### Immunofluorescence staining.

For immunofluorescence, cells seeded on the slides were washed with PBS 3 times and fixed with methanol or 4% paraformaldehyde (PFA) at −80°C overnight. Cells were permeabilized and blocked with PBS-TBH (10% FBS, 3% bovine serum albumin [BSA], 1× PBS) at room temperature (RT) for 30 min, incubated with primary antibodies at appropriate dilutions in PBS-TBH overnight at 4°C or 1 h at 37°C, and fluorophore-conjugated (Alexa Fluor Plus 488 and Alexa Fluor Plus 594 [Invitrogen; A32723 and A11012, respectively]) secondary antibodies at appropriate dilutions in PBS-TBH for 30 min at 37°C in the dark. The slides were then mounted with mounting medium with DAPI (4′,6-diamidino-2-phenylindole) (Abcam; ab104139) and photographed under a Carl Zeiss Axio Imager Z2.

### Subcellular fractionation.

For extraction of nuclear and cytoplasmic proteins, 1 × 10^6^ cells were collected by trypsin treatment or cell scraping, centrifuged at 1,000 × *g* for 5 min, resuspended in 0.5 mL of ice-cold buffer 1 (150 mM NaCl, 50 mM HEPES [pH 7.4], 25 μg/mL digitonin, 10 μL/mL protease inhibitor), incubated for 30 min at 4°C, and then centrifuged at 4,600 pm for 5 min. The supernatants represented the cytosol-enriched fraction. The pellets were then washed with 1 mL of ice-cold PBS and centrifuged at 4,600 rpm for 5 min to remove any remaining digitonin. The pellets were resuspended in 0.3 mL of ice-cold buffer 2 (150 mM NaCl, 50 mM HEPES [pH 7.4], 1% [vol/vol] NP-40, 10 μL/mL protease inhibitor) and incubated for 30 min on ice. The samples were centrifuged at 8,700 rpm for 5 min, and the supernatant represented the extract comprising membrane-bound organelles. The pellets were washed again with 1 mL ice-cold PBS and centrifuged at 8,700 rpm for 5 min to remove any remaining NP-40. The pellets were then resuspended in 0.2 mL of ice-cold buffer 3 (150 mM NaCl, 50 mM HEPES [pH 7.4], 0.5% [wt/vol] sodium deoxycholate, 0.5% [wt/vol] SDS, 1 mM dithiothreitol [DTT], 10 μL/mL protease inhibitor) and incubated at 4°C for 30 min followed by sonication twice (10 s, 20% amplification). The solution represented nuclear extract (62).

### Coimmunoprecipitation and immunoblotting.

For immunoprecipitation, the cells were collected and lysed in subcellular fractionation extraction buffer or radioimmunoprecipitation assay (RIPA) buffer. The extracts were incubated with anti-Flag magnetic beads for 8 h at 4°C on rotors. The resin was washed 5 times with PBST and denatured in 2× SDS-PAGE loading buffer. For immunoblot analysis, whole-cell extracts or subcellular fractions were subjected to SDS-PAGE, transferred onto polyvinylidene difluoride (PVDF) membranes, and blotted with the indicated antibodies.

### CUT&Tag assay.

The CUT&Tag assay was performed using the NovoNGS CUT&Tag 2.0 high-sensitivity kit (for Illumina) (Novoprotein Scientific Inc; N259-YH01-01A) as previously described (56). In brief, 1 × 10^5^ HEp-2 or HEp-2 cells transfected with Flag-Sp100A were harvested by trypsin treatment, centrifuged at 800 × *g* for 5 min at RT, precipitated by concanavalin A (ConA)-bound magnetic beads, and resuspended in 100 mL Dig-wash buffer (20 mM HEPES [pH 7.5], 150 mM NaCl, 0.5 mM spermidine, protease inhibitor cocktail, 0.05% digitonin) containing 2 mM EDTA and primary anti-Flag antibody at a 1:100 dilution overnight at 4°C. The beads were washed in Dig-wash buffer 3 times and incubated with goat anti-mouse secondary antibody (Abcam; ab6708) for 1 h at a dilution of 1:200. After incubation, the beads were washed 3 times in Dig-Hisalt buffer (0.05% digitonin, 20 mM HEPES [pH 7.5], 300 mM NaCl, 0.5 mM spermidine, protease inhibitor cocktail). Cells were incubated with protein A-Tn*5* transposase at 37°C for 1 h and washed 3 times in Dig-Hisalt buffer to remove unbound protein A-Tn*5*. Next, the cells were resuspended in 100 μL of tagmentation buffer (10 mM MgCl_2_ in Dig-Hi salt buffer) and incubated at 37°C for 1 h. The tagmentation was terminated by adding 2.25 mL of 0.5 M EDTA, 2.75 mL of 10% SDS and 0.5 mL of 20 mg/mL proteinase K at 55°C for 1 h. The DNA fragments were extracted by phenol chloroform and amplified using the provided barcode primers with 15 cycles of PCR. CUT&Tag libraries were cleaned with a single round of Novo NGS DNA clean beads (provided in the kit) at a 1.3 to 1 (vol/vol) ratio of beads to sample, quantified, and pooled for sequencing.

### DNA sequencing and data processing.

The size distribution and molar concentration of the libraries were determined using a Qsep instrument. Paired-end Illumina sequencing was performed on the barcoded libraries following the manufacturer’s instructions. Raw sequences were trimmed using FASTp 0.19.11 (63) with the options length required = 50 and n_base_limit = 6 to discard adapter and low-quality sequences. The clean sequences were then aligned to hg38 by BWA 0.7.12-r1039 with the following parameters: −T 25 −k 18. Only uniquely mapped sequences were extracted for downstream analyses (64). CUT&Tag signals in upstream and downstream 3-kb areas of transcriptional initiation sites (TSSs) were analyzed with the compute matrix module of deepTools software (version 3.0.2) (65). MACS2 2.1.2 was used to identify the peaks that were significantly enriched in the pairwise comparisons with the following parameters: *q* value (false-discovery rate) = 0.05, call summits, nomodel, shift = −100, extsize = 200, keep-dup all (66). Chipseeker software was used to analyze the distribution of peaks in different regions of the human genome (67).

### Animal experiments.

Four-week-old BALB/c mice (SPF Biotechnology Co., Ltd., Beijing, People’s Republic of China) were infected with 1 × 10^7^ PFU of VSV in 50 μL per mouse through the intranasal route. Mice were weighed on the day of infection (original weight) and every day postinfection. A humane endpoint of the experiment was reached when the infected animal lost more than 25% of its original body weight. Mice were sacrificed at the indicated time points postinfection, and lung samples were collected, weighed, and homogenized in 500 μL DMEM with 2% penicillin-streptomycin using a Qiagen TissueLyser (Qiagen; 85600). The mixture was centrifuged at 12,000 × *g* at 4°C for 15 min, and the supernatant was collected for IFN-β ELISA and virus titration in Vero cells. For RNA extraction and quantitative reverse transcription-PCR (qRT-PCR), lung samples were homogenized in 1 mL TRIzol per organ. Total RNA was extracted and reverse transcribed as previously described (16). The RNA levels of the tested ISGs and the VSV RNA genome were quantified by qRT-PCR using the primers listed in Table S3. All animals were kept in a pathogen-free environment and fed ad lib. The procedures for the care and use of animals were approved by the Ethics Committee of the School of Medicine of Sun Yat-sen University with the reference ID SYSU-IACUC-MED-2021-B0139. All applicable institutional and governmental regulations concerning the ethical use of animals were followed.

10.1128/mbio.02044-22.8 TABLE S3 qRT-PCR primers for human, mouse, and virus detection. Download Table S3, DOCX file, 0.02 MB. Copyright © 2022 Dong et al. 2022 Dong et al. https://creativecommons.org/licenses/by/4.0/ This content is distributed under the terms of the Creative Commons Attribution 4.0 International license.

### Statistical analysis.

In this study, each of the experiments was performed with at least three biological replicates unless otherwise specified. Data are presented as the mean ± standard deviation (SD) calculated by GraphPad Prism 6.0 software. Two-tailed unpaired Student's *t* test or ordinary one-way or two-way analysis of variance (ANOVA), as indicated in each figure, was used to calculate *P* values. Significance is indicated as follows: n.s., not significant (*P* > 0.05); *, *P* ≤ 0.05; **, *P* ≤ 0.01; ***, *P* ≤ 0.001; ****, *P* ≤ 0.0001.
