# Supplementary material for: PML Body Component Sp100A Is a Cytosolic Responder to IFN and Activator of Antiviral ISGs
Source: mBio. 2022 Nov 16;13(6):e02044-22. doi: 10.1128/mbio.02044-22 (PMC9765618; doi:10.1128/mbio.02044-22)
Supplement: TABLE S1 [file mbio.02044-22-s0005.docx]

**Supplementary Table 1 Mass Spec identified phosphorylation sites on cytosolic Sp100A.**

| **Nontreated** | **Predicted phospho sites** |  | **Mutants** |
| --- | --- | --- | --- |
| LNEcIsPVANEMNHLPAHSHDLQR | S6 | Common in both groups | **M2** |
| GFENVIHDKLPLQEsEEEEREER | S15 | Common in both groups | **M12** |
| THHNQASDIIVISSEDsEGSTDVDEPLEVFISAPR | S13/S14/S17 | Unique site | **M32** |
| VIGQDHDFSESsEEEAPAEASSGALR | S12 | Common in both groups | **M34** |
| SGLQLSLEQGTGENsFR | S15 | Common in both groups | **M16** |
| SEPVINNDNPLEsNDEK | S13 | Unique site | **M31** |
| LSTFREsFKK | S7 | Common in both groups | **M33** |
|  |  |  |  |
| **IFNB treated** |  |  |  |
| VIGQDHDFSESsEEEAPAEASSGALR | S12 | Common in both groups | **M34** |
| LNEcIsPVANEmNHLPAHSHDLQR | S6 | Common in both groups | **M2** |
| LSTFREsFK | S7 | Common in both groups | **M33** |
| GFENVIHDKLPLQEsEEEEREER | S15 | Common in both groups | **M12** |
| SLTWPPsGSPSHAGTTPPENGLSEHPcETEQINAK | S7/S9 | Unique site | **M17** |
| SGLQLSLEQGTGENsFR | S15 | Common in both groups | **M16** |
