# Supplementary material for: PML Body Component Sp100A Is a Cytosolic Responder to IFN and Activator of Antiviral ISGs
Source: mBio. 2022 Nov 16;13(6):e02044-22. doi: 10.1128/mbio.02044-22 (PMC9765618; doi:10.1128/mbio.02044-22)
Supplement: TEXT S1 [file mbio.02044-22-s0008.docx]

**Supplementary materials and methods**

**Antibodies and Reagents**

Information on the antibodies and reagents used in this study is as follows: rabbit polyclonal anti-Sp100 antibody (GeneTex, GTX131569), mouse monoclonal anti-Flag antibody (Abways, AB0008), rabbit polyclonal anti-Flag antibody (Proteintech, 80010), mouse monoclonal anti-β-actin antibody (Sino Biological, 1000166), mouse monoclonal anti-GAPDH antibody (Abways, AB0037), mouse monoclonal anti-histone antibody (Sino Biological, 100005), mouse monoclonal anti-PKM2 antibody (Abways, AB3215), rabbit polyclonal anti-PML antibody (Abcam, ab179466), mouse monoclonal anti-PML antibody (Santa Cruz, sc966), and rabbit polyclonal anti-HA antibody (CST, 3724s). Mouse monoclonal anti-IFI16 antibody (Santa Cruz, sc8023), Mouse monoclonal anti-RIG-I antibody (Santa Cruz, sc376845), Mouse monoclonal anti-GST antibody (Abways, AB0055), Mouse monoclonal anti-His antibody (Abways, AB0002), Anti-Flag Magnetic Beads (Biolinkedin, L-1011), Goat anti-mouse IgG antibody (Invitrogen, 31430), Goat anti-rabbit IgG antibody (Invitrogen, 32460), TRIzol reagent (Invitrogen, A33251), NeutraKine IFN beta monoclonal antibody (Proteintech, 69013), Rabbit polyclonal anti-PI3K P85/P55 antibody (Affinity biosciences, AF6242), Rabbit polyclonal anti-ERK 1/2 antibody (Affinity biosciences, AF0155), Rabbit polyclonal anti-pERK 1/2 antibody (Affinity biosciences, AF1015), Rabbit polyclonal anti-p-PI3K antibody (Affinity biosciences, AF3242), PI3K signaling pathway inhibitor GDC0941 (Selleck, S1065) was used at 10 μM, Protease inhibitor cocktail (Thermo Fisher scientific, EO0492), Cycloheximide (Selleck, NSC-185)(CHX) was used at 50 ug/ml, Recombinant Human IFN-beta Protein (IFNβ) (R&D system, 8499-IF-010/CF) was used at 1000 U/ml for all experiments.

**ELISA**

The production and secretion of human IFNβ in HEp-2 cell supernatants was assessed with a human IFNβ ELISA kit (MultiSciences, 70-EK1236-96). Mouse IFNβ kits (MultiSciences, 70-EK2236-96) were used for the detection of IFNβ in mouse samples.

**RNA Extraction and qRT-PCR**

RNA was extracted using a total RNA extraction kit (OMEGA, R6834) or TRIzol reagent following the manufacturer’s protocol. Reverse transcription was performed using an Evo M-MLV RT Kit for qPCR (Accurate Biology, AG11603). Gene expression was detected by real-time qPCR using Sybrgreen detection systems (Accurate Biology, AG11701). Primer information is listed in Supplementary Table 3.

**Immunofluorescence Staining**

For immunofluorescence, cells seeded on the slides were washed with PBS 3 times and fixed with methanol or 4% PFA at -80°C overnight. Cells were permeabilized and blocked with PBS-TBH (10% FBS, 3% bovine serum albumin (BSA), 1XPBS) at room temperature (RT) for 30 min, incubated with primary antibodies at appropriate dilutions in PBS-TBH overnight at 4°C or 1 hr at 37°C, and fluorophore conjugated (Alexa Fluor plus 488 (Invitrogen, A32723), Alexa Fluor plus 594 (Invitrogen, A11012)) secondary antibodies at appropriate dilutions in PBS-TBH for 30 min at 37°C in the dark. The slides were then mounted with mounting medium with DAPI (Abcam, ab104139) and photographed under a Carl Zeiss Axio Imager Z2.

**GST pull-down**

The E. coli BL21 strain (DE3) was transformed with the plasmids pET-28a-His-PKM2, pGEX4T-1-GST or pGEX4T-1-GST-Sp100A. Bacterial-induced recombinant proteins were purified using Ni-Sepharose beads or GST-Sepharose beads as described previously(1). To pull down cytosolic Sp100A-interacting proteins, purified GST or GST-Sp100A was incubated with cytosolic fractions of nontreated or IFN-treated HEp-2 cells for 12 hr at 4°C. The glutathione Sepharose mixture was collected and washed 3 times with PBST, denatured in 2X SDS loading buffer, separated in a 10% polyacrylamide gel, and silver stained according to the manufacturer’s protocol (Sangon, C500021). To confirm the direct interaction between GST-Sp100A and His-PKM2, anti-GST antibody and glutathione sepharose (GE Healthcare, 17513201) were incubated at 4 °C for 2 hr in PBST, washed, and then incubated with bacterial purified GST-Sp100A and His-PKM2 at 4 °C for 2 hr. The final precipitates were collected, washed three times, denatured, and analyzed by immunoblotting using anti-GST antibody and anti-His antibody.

**Infection**

HEp-2 cells or other cell lines at 90% confluency were inoculated with VSV-GFP or VSV-Sp100A diluted in DMEM at a desired multiplicity of infection (MOI) at 37°C for 2 hr, and the inoculum was replaced by 1% FBS DMEM. The culture medium was collected at the indicated time points post infection, and the virus concentration was titrated in Vero cells by plaque assay.

**ATP depletion**

ATP depletion in HEp-2 cells was performed as previously described (2). In brief, HEp-2 cells were washed with PBS and cultured in glucose-free DMEM (Gibco, 11966) containing penicillin (100 U/ml), streptomycin sulfate (100 g/ml), HEPES (10 mM, pH 7.3), and 10% FBS for 12 hr. The culture medium was then replaced with ATP depletion medium (glucose-free media, 10 mM sodium azide, 6 mM 2-deoxy-D-glucose (Sigma, D8375)) plus IFNβ at 1000 U/ml for 2 hr. The subcellular distribution of Sp100 was investigated by immunoblotting.

**H&E staining**

For hematoxylin & eosin (H&E) staining of lung sections, mice were sacrificed and fixed via cardiac perfusion with 4% paraformaldehyde after flushing out the red blood cells with 0.1 M PBS. Fixed lung samples were dehydrated in a graded alcohol series, cleared in xylene, and embedded in paraffin wax. The embedded lung tissues were cut into sections (5 µm), mounted on glass slides, and stained with hematoxylin & eosin (H&E). Stained tissue sections were photographed under a light microscope (Olympus-IX 51) for histological analysis.

**References**

1. S. Li *et al.*, The tumor suppressor PTEN has a critical role in antiviral innate immunity. *Nat Immunol* **17**, 241-249 (2016).

2. E. D. Schwoebel, T. H. Ho, M. S. Moore, The mechanism of inhibition of Ran-dependent nuclear transport by cellular ATP depletion. *The Journal of cell biology* **157**, 963-974 (2002).
