# Supplementary material for: PML Body Component Sp100A Is a Cytosolic Responder to IFN and Activator of Antiviral ISGs
Source: mBio. 2022 Nov 16;13(6):e02044-22. doi: 10.1128/mbio.02044-22 (PMC9765618; doi:10.1128/mbio.02044-22)
Supplement: TABLE S2 [file mbio.02044-22-s0006.docx]

**Supplementary Table 2. Primers for plasmid and mutation construction.**

| Gene | Forward | Reverse |
| --- | --- | --- |
| Flag-SP100C | AACTTAAGCTTGCCACCATGGACTACAAAGACGATGACGACAAGGCAGGTGGGGGCGGCGACCTGAGCAC | CTAGGCGATATCCCGCTAAATAAACATTATAATG |
| Flag-SP100HMG | AACTTAAGCTTGCCACCATGGACTACAAAGACGATGACGACAAGGCAGGTGGGGGCGGCGACCTGAGCAC | TTAGGCGATATCCCGTTATTTATCATCATCTTCTTCA |
| Flag-SP100B | AACTTAAGCTTGCCACCATGGACTACAAAGACGATGACGACAAGGCAGGTGGGGGCGGCGACCTGAGCAC | GCGTTAGATATCCGCTCACTTGATCATCACCTT |
| Flag-SP100A | AACTTAAGCTTGCCACCATGGACTACAAAGACGATGACGACAAGGCAGGTGGGGGCGGCGACCTGAGCAC | TGTGCTGGATATCCGCCTAATCTTCTTTACCTGACCCTCTTC |
| HA-PKM2 | AATTGCTAGCGCCATGTCGAAGCCCCATAGTGA | AATTGCGGCCGCCGGCACAGGAACAACACGCAT |
| Scr shRNA | CCGGAAAAACAGTGTACTTTACCAATATACTCGAGTATATTGGTAAAGTACACTG | CAGTGTACTTTACCAATATACTCGAGTATATTGGTAAAGTACACTGTTTTTAATT |
| Sp100A-shRNA-1 | CCGGGAGGGTCAGGTAAAGAAGATTCTCGAGAATCTTCTTTACCTGACCCTCTTTTTG | AATTCAAAAAGAGGGTCAGGTAAAGAAGATTCTCGAGAATCTTCTTTACCTGACCCTC |
| Sp100A-shRNA-2 | CCGGGCCTGCAGAATGTCAGGAATGCTCGAGCATTCCTGACATTCTGCAGGCTTTTTG | AATTCAAAAAGCCTGCAGAATGTCAGGAATGCTCGAGCATTCCTGACATTCTGCAGGC |
| MUT188(S-A) | GGAACTGGTGAAAACGCATTTCGAAGCCTGACTT | CCAAGTCAGGCTTCGAAATGCGTTTTCACCAGTTC |
| MUT188（M16）(S-D) | GGGGTGAAAACGATTTTCGAAGCCTGACTTGGC | CGCTTCGAAAATCGTTTTCACCAGTTCCTTGTTC |
| MUT197 (M17) (S-A) | TTGGCCACCTGCGGGTTCCCCATCTCAT | ATGAGATGGGGAACCCGCAGGTGGCCAA |
| MUT2 (M2) (S-A) | GGAGGCTGAATGAATGTATTGCTCCAGTAG | CATTTGCTACTGGAGCAATACATTCATTCAGCC |
| MUT12 (M12) (S-A) | AATTGCCTCTCCAAGAAGCAGAAGAAGAAG | TCCCTCTCTTCTTCTTCTGCTTCTTGGAGA |
| ShPKM2-1 | CCGGGCTGTGGCTCTAGACACTAAACTCGAGTTTAGTGTCTAGAGCCACAGCTTTTTG | AATTCAAAAAGCTGTGGCTCTAGACACTAAACTCGAGTTTAGTGTCTAGAGCCACAGC |
| ShPKM2-2 | CCGGGTTCGGAGGTTTGATGAAATCCTCGAGGATTTCATCAAACCTCCGAACTTTTTG | AATTCAAAAAGTTCGGAGGTTTGATGAAATCCTCGAGGATTTCATCAAACCTCCGAAC |
| ShIFNAR | CCGGAAGAACTACAGCAGGACTTTGCTCGAGCAAAGTCCTGCTGTAGTTCTTTTTTTG | AATTCAAAAAAAGAACTACAGCAGGACTTTGCTCGAGCAAAGTCCTGCTGTAGTTCTT |
| Mycoplasma 1 | ACACCATGGGAGCTGGTAAT | CTTCATCGACTTTCAGACCCAAGGCAT |
| Mycoplasma 2 | GTTCTTTGAAAACTGAAT | GCATCCACCAAAAACTCT |
