# Supplementary material for: PML Body Component Sp100A Is a Cytosolic Responder to IFN and Activator of Antiviral ISGs
Source: mBio. 2022 Nov 16;13(6):e02044-22. doi: 10.1128/mbio.02044-22 (PMC9765618; doi:10.1128/mbio.02044-22)
Supplement: TABLE S3 [file mbio.02044-22-s0007.docx]

**Supplementary Table 3. qRT-PCR primers for human, mouse, and virus detection.**

| Human primers | | |
| --- | --- | --- |
| Gene | Forward | Reverse |
| NUP98 promoter | CTGGGTCGACCCTGTTATTC | TCTCACCGCTCCTACCCTGT |
| DDX58 promoter | CAGTTGGCTAACTGGGACAT | GGGTTGAAGGGACACAGAGC |
| OAS2 promoter | GAGCCTCTGAGCTTAAGT | GCCCAGAGCCAGGAAACTGA |
| ISG15 promoter | TTTGTTTCTTCCGCTCACTCTG | AAGCCTGAGGCACACACGTC |
| IFI16 promoter | TGTCCCCAAATCTCATGCTG | GAGCCATTCATGAGGGATCC |
| hOAS2 | AACTGCTTCCGACAATCAAC | CCTCCTTCTCCCTCCAAAA |
| hMX2 | AGTCTTCGGTTTCCTCCTTTA | CTGCAAGGAGTCACCATTCT |
| hRIG-I | GGACGTGGCAAAACAAATCAG | GCAATGTCAATGCCTTCATCA |
| hISG 15 | GTGGACAAATGCGACGAACC | TCGAAGGTCAGCCAGAACAG |
| hISG 54 | ACGGTATGCTTGGAACGATTG | AACCCAGAGTGTGGCTGATG |
| hIFI16 | AAAGTTCCGAGGTGATGC | TGACAGTGCTGCTTGTGG |
| hGAPDH | GAAGGTGAAGGTCGGAGTC | GAAGATGGTGATGGGATTTC |
| hIFNβ | GCCGCATTGACCATCTATGAGA | GAGATCTTCAGTTTCGGAGGTAAC |
| hC-MYC | GGCTCCTGGCAAAAGGTCA | CTGCGTAGTTGTGCTGATGT |
| SP100-C | GTTGACCCTTGTGAGGAGCAT | TGTCCGCCTTTGCCATATCTT |
| SP100-HMG | CTGCCTGAGGAGCAGTTGAA | CGGTTCTGAGGCGAAAAAGC |
| SP100-B | TCTGCCAATGTCTCGTCTATTATGT | TTATGATGATGGGTCAATTTAAAGACTGT |
| SP100-A | ACTTGGCCTGCAGAATGTCA | CAAGGTAGTGAAGGTGCTCAGA |
| Mouse qPCR primers | | |
| Gene | Forward | Reverse |
| mOAS2 | TAGACCAGGCCGTGGATG | GTTTCCCGGCCATAGGAG |
| mRIG-I | GCCCTGTACCATGCAGGTTAC | AGTCCCAACTTTCGATGGCTT |
| mIFI16 | TCAGTTTCAGTAGCCACGGTAGCA | TGGTCCCAAACAAGTGATGGTGC |
| 18S | GGACCAGAGCGAAAGCATTTGCC | TCAATCTCGGGTGGCTGAACGC |
| Virus qPCR primers | | |
| Gene | Forward | Reverse |
| VSV-N | ACGGCGTACTTCCAGATGG | CGACCTTCTGGCACAAGAG |
| DENV2 | CAATATGCTGAAACGCGAGAGAAA | AAGACATTGATGGCTTTTGA |
| PR8-NP | AGGACAAGAGCTCTTGTTCG | CTCTTGTGTGCTGGATTCTC |
| PR8-HA | TGAACTATTACTGGACCTTGCT | CTCCTATTGTGACTGGGTGTAT |
| ZIKV | TTGGTCATGATACTGCTGATTGC | CCTTCCACAAAGTCCCTATTGC |
| SiRNAs | | |
| Target genes | Name | Sequence(5’-3’) |
| Human Pin-1 | Pin1 kd1 | CGAGGCGUCUUCAAAUGGCTT |
| Human Pin-1 | Pin1 kd2 | CGCUGAACUGUGAGGCCAGTT |
| Human Importin-α | Importin-α siRNA-1 | AUAACCAGAAUAAUCUUGGTT |
| Human Importin-α | Importin-α siRNA-2 | UAAGGUAGGAAGAAUCUGCTT |
| Human Importin-β | Importin-β siRNA-1 | ACAUUCUGAAGAGUUGCACTT |
| Human Importin-β | Importin-β siRNA-2 | AUCAAUAGCAAGCCACCUCTT |
| Human PKM2 | PKM2 siRNA | CCAUAAUCGUCCUCACCAA |
| Negative control | Scramble siRNA | UUCUCCGAACGUGUCACGUTT |
